# Effects of legume kernel fibres and citrus fibre on putative risk factors for colorectal cancer: a randomised, double-blind, crossover human intervention trial

**DOI:** 10.1186/1475-2891-12-101

**Published:** 2013-07-16

**Authors:** Anita Fechner, Katrin Fenske, Gerhard Jahreis

**Affiliations:** 1 Department of Nutritional Physiology, Friedrich Schiller University of Jena, Dornburger Straße 24, D-07743 Jena, Germany

**Keywords:** Dietary fibre, Lupin fibre, Soya fibre, Citrus fibre, Bile acids, Short-chain fatty acids, Colorectal cancer, Human study

## Abstract

**Background:**

In some studies, high intake of dietary fibre has been associated with a lower risk of colorectal cancer. The present study aimed to compare physiological effects of three legume kernel fibres and citrus fibre on blood lipids (primary outcome: LDL cholesterol) and colonic health.

**Methods:**

Ninety-two subjects were recruited for the double-blind, controlled crossover trial. Seventy-eight participants were randomly divided into three groups. Following run-in, half the volunteers from each group consumed 25 g/d of a legume fibre, comprising blue lupin fibre, white lupin fibre, and soya fibre for two weeks. The other half received the same amount of citrus fibre (active comparator). The intervention was crossed within each group after two weeks wash-out. At the end of run-in and intervention, a quantitative faeces collection took place and fasting blood samples were drawn. Repeated measures ANOVA with the general linear model were applied to evaluate changes following interventions.

**Results:**

Seventy-six subjects completed the study. Dietary fibre intake during all interventions was approximately twice the fibre intake at run-in. The lupin fibre supplementations increased daily faecal dry matter and faecal weight compared to run-in, representing an increase of 1.76 g faeces/g additional dietary fibre contributed by blue lupin and of 1.64 g faeces/g by white lupin, respectively. Both lupin interventions led to a significantly enhanced formation of short-chain fatty acids, and blue lupin fibre to a decrease in faecal pH compared to run-in (0.27 units, *P* < 0.01). Further, blue lupin increased primary bile acids-excretion (*P* = 0.02). All legume fibres reduced faecal concentrations of total and secondary bile acids (blue lupin: 16%; white lupin: 24%; soya: 16%). Blood lipids were not influenced by any intervention. No serious adverse effects were observed.

**Conclusions:**

The tested fibre preparations do not affect lipid metabolism through bile acid-binding in normocholesterolaemic subjects. However, particularly blue lupin kernel fibre improve colonic function and have beneficial effects on putative risk factors for colorectal cancer such as faecal mass, transit time, SCFA, faecal pH, and secondary bile acid concentration. Therefore, enhancing dietary fibre intake through blue lupin up to about 50 g/d can be recommended.

**Trial registration:**

NCT01036308

## Background

Colorectal cancer is the second most common cause of death from cancer, with 447,000 new cases diagnosed in Europe in 2012 [1]. Nutritional factors are thought to be responsible for an estimated 50% of the incidence [2]. A high intake of dietary fibre has been associated in some, but not all, studies with a lower risk of colorectal cancer. Therefore, supplementation with dietary fibre is of special importance as far too little fibre is generally consumed. Current guidelines from the European Food Safety Authority recommend an intake of 25 g of dietary fibre per day (2 g/MJ) for adults [3]. Many epidemiological studies support Burkitt’s hypothesis that increased dietary fibre intake leads to a reduced risk of colorectal cancer [4-6], while others revealed contradictory effects [7,8]. Case–control studies have generally shown a protective association [9], whereas results from cohort studies have not been consistent [4,8,10-14]. The inconsistencies of study findings may in part be explained by heterogeneity regarding the chemical and physicochemical properties of dietary fibre and the source of fibre [6,7,15,16]. However, the World Cancer Research Fund (WCRF), in conjunction with the American Institute for Cancer Research (AICR), released an updated meta-analysis of 25 prospective studies in 2011, which concludes that 10 g/d of total dietary fibre decreases the relative risk of colorectal cancer by 10% [2,17].

Legume seeds are a valuable plant source of dietary fibre. Besides favourable technofunctional properties, legume kernel fibres offer several physiological benefits [18-21]. There is evidence indicating that consumption of legume kernel fibre, *e.g.* soya and lupin kernel fibre, may have a preventive impact on colorectal cancer [18,19]. Aune *et al.* reported a 38% reduction in relative risk for each 10 g/d intake of legume fibre [16].

Several plausible mechanisms for the protective effects of dietary fibre on colorectal cancer have been hypothesised. These include direct effects on the composition of the gut and on bowel habits together with indirect effects such as systemic changes in insulin and hormonal exposures [4,7,16,22,23]. In particular, dietary fibre benefits colonic health by decreasing the toxicity of colonic content. Fibre, which is not soluble in water, enhances faecal bulk by its ability to bind water [24]. The increased gut content promotes stimulation of intestinal peristalsis and reduction of transit time which leads to a decreased contact of enterocytes with harmful substances, particularly carcinogens [15,24]. Water-soluble fibre is known to have less effect on faecal bulking because they are largely fermented by resident bacteria in the colon. The fermentation process promotes bacterial growth and produces short-chain fatty acids (SCFA) in the colon [15]. SCFA are an important energy source for colonocytes. Moreover, *n*-butyrate is able to reduce the risk of malignant changes through regulation of colonocyte differentiation [25,26]. Further, SCFA lower the pH value of the colon resulting in decreased formation of carcinogens from bacterially degraded bile acids and cholesterol, and restricted growth of potentially pathogenic bacteria [24,25,27]. Dietary fibre is also thought to modulate faecal concentration, distribution and excretion of bile acids due to bile acid binding, increased faecal bulk and altered pH value. Bile acids, in particular, secondary bile acids are putative risk factors for colorectal cancer [28,29].

Moreover, the ability of fibre, in particular the water-soluble fraction, to bind bile acids possibly leads to an interruption of the enterohepatic circulation resulting in lower LDL cholesterol concentrations in blood [30].

Thus, this double-blind crossover intervention trial was designed to compare broad physiological effects of specific types of legume fibre with citrus fibre. In particular, the study aimed to evaluate the short-term effects of three isolated legume kernel fibre preparations on blood lipids (primary outcome: LDL cholesterol), markers of intestinal health, bowel function and in relation to colorectal carcinogenesis in healthy men and women.

## Methods

### Fibre preparations

Legume kernel fibre preparations from *Lupinus angustifolius cv.* Boregine (blue lupin), *Lupinus albus cv.* Typ Top (white lupin), and *Glycine max cv.* Hefeng (soya) were produced and provided by the Fraunhofer Institute for Process Engineering and Packaging (Fh-IVV, Freising, Germany). Legume seeds were cleaned, dehulled and separated into hulls and kernels by sifting. The kernels were then flaked and deoiled. Extraction of proteins and other water-soluble substances from kernels was followed by pasteurisation. The fibre preparations were then lyophilised and grounded to a fine powder [21,31].

The citrus fibre (Herbacel AQ Plus) provided by Herbafood (Herbafood Ingredients GmbH, Werder, Germany) was chosen as active comparator (citrus) due to its similar composition and comparable properties. Herbacel AQ Plus is made from de-oiled, dried citrus fruits. Non-fibrous compounds such as sugars, colouring and flavouring components are removed during numerous washing steps. This results in a product with neutral sensory properties as well as a high water-binding and retention capacity. The nutrient composition of the fibre preparations (Table 1) was analysed using the standard methods according to van Soest *et al.*[32] and the Association of Official Analytical Chemists [33]. All experimental fibres contained both soluble and insoluble fibre fractions. The insoluble fibre portion comprised of hemicellulose and cellulose. The fraction of soluble fibre in both lupin preparations was markedly higher than in soya or citrus fibre. In addition, the soluble fibre levels of both lupin preparations were slightly higher than the value of about 45% previously reported [19,34], which may be associated to the modified processing method [21,31].

**Table 1:** Compositional data of fibre preparations used in the human intervention study

	**Blue lupin**	**White lupin**	**Soya**	**Citrus**
**Dry matter** [g/100 g FM]	92.5 ± 0.1	90.4 ± 0.2	91.1 ± 0.2	91.1 ± 0.1
**Ash** [g/100 g DM]	1.67 ± 0.14	1.71 ± 0.05	2.45 ± 0.24	1.34 ± 0.12
**Protein** [g/100 g DM]	9.68 ± 0.51	12.4 ± 1.1	18.2 ± 0.1	5.31 ± 0.14
**Fat** [g/100 g DM]	1.12 ± 0.10	1.31 ± 0.12	1.49 ± 0.19	0.31 ± 0.13
**Total dietary fibre** [g/100 g DM]	86.7 ± 2.1	83.4 ± 2.8	77.3 ± 1.0	92.2 ± 0.2
**Soluble dietary fibre** [g/100 g DM]	52.6 ± 2.1	50.9 ± 2.8	33.2 ± 1.0	22.5 ± 0.2
**Insoluble dietary fibre** [g/100 g DM]	34.1 ± 1.5	32.5 ± 2.1	44.1 ± 0.9	69.7 ± 0.3
**Hemicellulose** [g/100 g DM]	10.9 ± 1.5	16.4 ± 2.1	21.7 ± 0.9	3.70 ± 0.3
**Cellulose** [g/100 g DM]	23.2 ± 0.9	16.1 ± 1.4	22.4 ± 1.1	65.8 ± 1.2
**Lignin** [g/100 g DM]	0	0	0	0.22 ± 0.31

Values are presented as mean ± standard deviation.

Blue lupin, *Lupinus angustifolius cv.* Boregine*;* white lupin, *Lupinus albus cv.* Typ Top*;* soya, *Glycine max cv.* Hefeng*;* citrus, citrus fibre Herbacel AQ Plus serving as active comparator; *FM* fresh matter, *DM* dry matter.

Currently, fibre preparations of lupin, in particular blue lupin, soya and citrus are used worldwide serving as functional additive in various food products, including *e.g.* bakery, milk and meat products.

### Subjects

**Figure 1:**
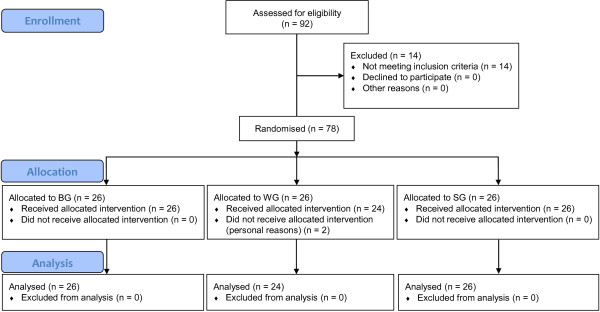
**CONSORT flow diagram.** BG, blue lupin group; WG, white lupin group; SG, soya group; blue lupin, *Lupinus angustifolius cv.* Boregine; white lupin, *Lupinus albus cv.* Typ Top; soya, *Glycine max cv.* Hefeng.

The study was conducted at the Friedrich Schiller University Jena (Institute of Nutrition, Department of Nutritional Physiology, Jena, Germany). A total of 92 healthy subjects were recruited for the study by advertising in newspapers and posting notices on bulletin boards in university institutes. To be eligible, participants had to be between 20 and 45 years of age, and in good physical health. Exclusion criteria for all participants included: legume allergy, milk or lactose intolerance, chronic diseases, pregnancy, lactation, and intake of pharmaceuticals and nutritional supplements. Seventy-eight volunteers fulfilled the selection criteria and were randomly assigned to three groups *via* group matching. Participant flow throughout the trial is presented in Figure 1. Volunteers were provided with detailed information regarding purpose, course, and possible risks involved in the study. A written informed consent was obtained from all participants. The study was approved by the Ethical Committee of the Medical Faculty of the Friedrich Schiller University Jena (1864-09/06). The consort checklist can be found in Additional file 1.

### Study design

**Figure 2:**
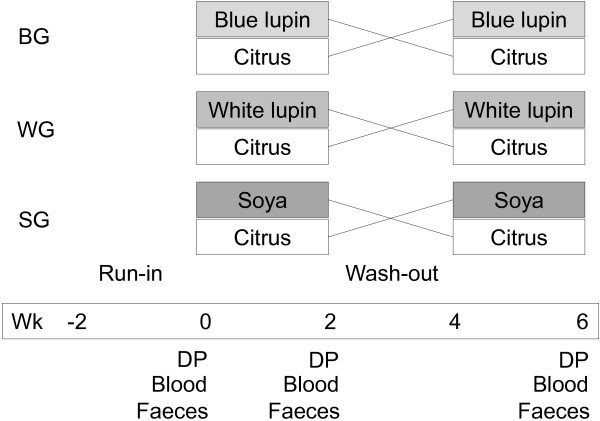
**Study design.** BG, blue lupin group; WG, white lupin group; SG, soya group; blue lupin, *Lupinus angustifolius cv.* Boregine; white lupin, *Lupinus albus cv.* Typ Top; soya, *Glycine max cv.* Hefeng; citrus, citrus fibre Herbacel AQ Plus serving as active comparator; wk, week; DP, dietary protocol (last five days); blood, blood sampling after overnight fasting (last day); faeces, 3-d collection of faeces (last three days).

The study was designed as a double-blind, controlled, randomised crossover trial to ensure that each volunteer served as his or her own control. All persons involved in the study including scientific staff and study participants were blinded. In addition, study products were blinded and labelled using a numeric code. The participants were randomly divided into three groups: i. the blue lupin group (BG, n = 26) consumed a fibre preparation of *Lupinus angustifolius cv.* Boregine (blue lupin), ii. the white lupin group (WG, n = 26) received *Lupinus albus cv.* Typ Top fibre (white lupin), and iii. the soya group (SG, n = 26) ingested *Glycine max cv.* Hefeng fibre (soya). The conducted study was divided into four periods, each of two weeks duration: a run-in period, two intervention periods with supplementation of 25 g total dietary fibre per day as a legume fibre preparation or citrus fibre additionally to their usual diet, and a wash-out period between the intervention periods. At the beginning of the run-in period, participants were advised to maintain their lifestyle and nutritional habits throughout the study. During the run-in and wash-out periods, each subject included 150 g of a pure milk product (milk or yoghurt, 3.5% fat) of their own choice and 150 ml juice (apple, peach, banana, or pear) into their usual diet to exclude the physiological effects of these food products from the effects following the fibre interventions. The participants had to consume the same type and quantity of these foods during the intervention periods with added fibre. Throughout the first intervention period, half of the participants from each group received one of the legume fibre preparations (blue lupin, white lupin or soya) and the other half the citrus fibre (serving as active comparator) for two weeks. Of the daily fibre dose, half was stirred in the selected milk product and the other half in juice. After the wash-out period, intervention with legume fibre and the active comparator was crossed within each group and the respective fibre preparations were consumed for another two weeks. At the last five days of run-in period and of both intervention periods each subject had to record his or her individual requirements in a dietary protocol to determine their dietary fibre intake. Data were interpreted using PC-software PRODI® 5.0 expert (NutriScience GmbH, Freiburg, Germany). During the last three days of the run-in period and in both intervention periods, a quantitative faeces collection took place. After defaecation, the samples were transported directly to the institute and stored at -20°C. Fasting blood samples were drawn on the last day of each period (Figure 2). In addition, defaecation discomfort (flatulence, constipation) was assessed in records during the whole study. At the end of the study, volunteers had to document their compliance with the study protocol in an anonymous questionnaire.

The primary outcome of the study was the determination of LDL cholesterol concentration in serum. Secondary outcomes included estimation of blood lipids, general excretion parameters, and faecal concentration or excretion of SCFA, neutral sterols, bile acids and fibre.

### Faecal sample preparation

At the end of each period faecal specimens from all subjects were defrosted, homogenised, and aliquots were stored at -20°C. After homogenisation, faecal pH value was measured using a glass pH electrode (InLab 420 electrode, MP 225; Mettler Toledo GmbH, Giessen, Germany). One aliquot portion was used for analysis of SCFA and another one was freeze-dried for determination of neutral sterols, bile acids, and total food-derived fibre. For visual assessment of faecal consistency, the ‘Bristol Stool Form Scale’ (type 1 to 7) was used [35]. The oro-faecal transit time was determined *via* marker tablets containing carmine (E120; C.E. Roeper GmbH, Hamburg, Germany). After the first defaecation in each period, the subjects were required to ingest two carmine tablets and to document the time of intake together with the time of appearance of the red colour in faeces. The calculation of the transit time was repeated three times per period.

### Total dietary fibre

The concentrations of total dietary fibre in fibre preparations and the content of total food-derived fibre in freeze-dried faeces were determined by standard procedures recommended by the Association of Official Analytical Chemists [33] using the enzyme set BIOQUANT® Total Dietary Fibre (Merck, Darmstadt, Germany) and the filter machinery FIBERTEC E® described elsewhere [36].

### Short-chain fatty acids

Short-chain fatty acid (SCFA) analysis was carried out *via* gas chromatography-flame ionisation (Shimadzu-GC 17A; Shimadzu, Kyoto, Japan) using a ZB-FFAP column (15 m × 0.25 mm × 0.25 μm) as described previously [37]. After adding distilled water to fresh faeces, samples were mixed and centrifuged. Subsequently, the supernatant was added to the internal standard (*iso*-caproic acid). Before injection onto the column, the solution was mixed and centrifuged once more.

### Faecal neutral sterols and bile acids

Faecal neutral sterols and bile acids were analysed as described by Keller and Jahreis [38]. Briefly, aliquots of lyophilised faeces were transferred into a reaction tube containing the internal standard 5α-cholestane. The extraction of sterols was prepared with cyclohexane after a mild alkaline hydrolysis with ethanolic NaOH. The solvent was evaporated and extracts were reconstituted in decane, and injected in the gas chromatography-mass spectrometer (GC17-QP5000; Shimadzu, Kyoto, Japan) in duplicate. The total neutral sterol amount was defined as the sum of cholesterol, coprostanol, coprostanone, cholestanol and cholestanone. The sum of sterols without cholesterol was defined as metabolites [39]. The metabolic conversion rate was calculated as the proportion of total neutral sterols to metabolites.

For bile acid determination (primary: cholic acid, chenodeoxycholic acid; secondary: *iso*-lithocholic acid, lithocholic acid, *iso*-deoxycholic acid, deoxycholic acid, 12-ketodeoxycholic acid), the internal standard hyodeoxycholic acid was added to the aqueous phase of sterol extraction. After saponification with NaOH and acidification to pH 1.0 with HCl, bile acids were extracted with diethyl ether and evaporated. The residue was methylated and silylated. Following evaporation, the residue was dissolved in decane and the solution was injected into the gas chromatography-mass spectrometer (GC17-QP5000; Shimadzu, Kyoto, Japan).

### Blood lipids

Venous blood samples were taken from subjects after overnight fasting in serum monovettes. The samples were centrifuged (2500 × *g*, 20°C, 15 min) and the serum supernatant was stored at -80°C until analysis. Serum was analysed for total cholesterol, HDL cholesterol, and triacylglyceroles using commercially available kits (Beckmann, Krefeld, Germany) based on enzymatical tests and employing a fully automated analyser (Synchron LX, Beckmann Coulter, Fullerton, USA). The concentrations of LDL cholesterol were calculated using the Friedewald formula.

### Statistical analyses

Prior to the commencement of the study, a power analysis was performed using PASS 6.0 (NCSS Statistical Software, Kaysville, UT, USA) to evaluate sample size, based on data from the literature (LDL cholesterol). It resulted in a power of > 80% for this study. Samples of each participant were coded to protect volunteer identity and to mask treatment groups during the analysis. Statistical calculations were conducted using PASW Statistics version 18.0 (SPSS Inc., Chicago, IL, USA). Consistent with the requirement for analysis of variance (ANOVA), all data were checked for variance homogeneity and for normal distribution by applying the Levene’s test and the Kolmogorov-Smirnov test, respectively. To evaluate data in terms of statistical significance, repeated measures ANOVA with the general linear model were applied to identify changes in parameters over time. This model considers both individual and inter-individual changes. The intervention sequence as a consequence of the crossover design was considered by pasting the sequence as covariate. No influence of sequence was observed for any of the analysed parameters. Data that were not normally distributed were analysed using Wilcoxon signed-rank test to evaluate changes over time. For each comparison, *P* ≤ 0.05 was considered as statistically significant. Relationships between normally distributed variables were examined by calculating Pearson’s correlation coefficients; otherwise Spearman (^s^) correlation was used. Significant differences between periods are indicated by unequal superscripts. All results in text and tables are expressed as mean ± standard deviation.

## Results

### Initial characteristics, dietary fibre intake and compliance

Study dropout rate was 3% as two subjects (WG) withdrew from the study during the first week for personal reasons (Figure 1). Seventy-six volunteers (55 women, 21 men) with a mean age of 24.4 ± 3.2 years (range: 21 - 29 y) and a mean BMI of 21.7 ± 2.4 kg/m^2^ (range: 17.2 - 28.9 kg/m^2^) completed the trial successfully. The initial characteristics of subjects completing the study are shown in Table 2.

**Table 2:** Initial characteristics and blood lipids of subjects

	**BG (n = 26)**	**WG (n = 24)**	**SG (n = 26)**
**Age** [y]	25.6 ± 4.6	24.0 ± 2.2	23.8 ± 1.8
**Height** [cm]	172 ± 7	172 ± 8	170 ± 10
**Weight** [kg]	64.7 ± 11.9	66.9 ± 11.7	65.4 ± 10.9
**BMI** [kg/m^2^]	21.7 ± 2.5	22.5 ± 2.7	22.4 ± 2.0
**Total cholesterol** [mmol/L]	4.97 ± 0.93	4.99 ± 1.04	4.54 ± 1.13
**HDL cholesterol** [mmol/L]	1.64 ± 0.44	1.60 ± 0.40	1.47 ± 0.49
**LDL cholesterol** [mmol/L]	2.74 ± 0.70	2.78 ± 0.81	2.50 ± 0.69
**Triacylglyceroles** [mmol/L]	1.12 ± 0.44	1.00 ± 0.41	1.13 ± 0.47

Values are presented as mean ± standard deviation.

*BG* blue lupin group, *WG* white lupin group, *SG* soya group; blue lupin, *Lupinus angustifolius cv.* Boregine; white lupin, *Lupinus albus cv.* Typ Top; soya, *Glycine max cv.* Hefeng.

Consumption of the fibre preparations was well tolerated by most of the participants. The general compliance to the study protocol and the consumption of the recommended amount of the fibre preparations were indicated due to statements from anonymous questionnaires. Thus, the dietary fibre intake throughout the interventions was additional to their habitual fibre intake, which changed only slightly. Therefore, the total dietary fibre intake during all intervention periods was approximately twice the fibre intake at run-in (42.7 - 46.3 g/d; *P* < 0.01; Table 3).

**Table 3:** Dietary fibre intake, faecal composition and bowel habits

	**BG (n = 26)**			**WG (n = 24)**			**SG (n = 26)**		
	**Run-in**	**Blue lupin**	**Citrus**	**Run-in**	**White lupin**	**Citrus**	**Run-in**	**Soya**	**Citrus**
**Dietary fibre intake [g/d]**	22.4 ± 6.4^b^	45.6 ± 5.8^a^	46.1 ± 8.7^a^	18.4 ± 5.0^b^	42.7 ± 6.1^a^	43.3 ± 5.1^a^	21.8 ± 5.7^b^	46.3 ± 5.3^a^	45.3 ± 5.5^a^
**Faecal weight [g/d]**	132 ± 60^b^	176 ± 81^a^	171 ± 107^a^	103 ± 62^b^	144 ± 81^a^	142 ±65^a^	135 ± 63^b^	156 ±73^ab^	173 ± 70^a^
**Faecal dry matter**									
**[g/100 g]**	27.1 ± 5.1^a^	24.7 ± 5.3^b^	24.8 ± 5.0^b^	28.6 ± 5.8^a^	26.0 ± 5.3^b^	25.1 ± 4.4^b^	26.1 ± 5.0	25.6 ± 5.1	24.9 ± 4.7
**[g/d]**	34.2 ± 12.6^b^	40.7 ± 14.1^a^	38.4 ± 16.9^a^	28.2 ± 12.4^b^	35.7 ± 17.3^a^	35.0 ± 13.3^a^	33.6 ± 13.3^b^	38.2 ± 15.2^ab^	42.9 ± 18.9^a^
**Faecal pH**	6.65 ± 0.41^a^	6.38 ± 0.47^b^	6.49 ± 0.52^ab^	6.51 ± 0.33	6.54 ± 0.40	6.46 ± 0.40	6.42 ± 0.34	6.45 ± 0.30	6.45 ± 0.56
**Transit time [d]**^**1**^	1.29 ± 0.61^b^	1.02 ± 0.33^c^	1.48 ± 0.72^a^	1.47 ± 0.7	1.39 ± 0.57	1.28 ± 0.49	1.28 ± 0.61	1.33 ± 0.64	1.31 ± 0.42
**Bristol Stool Form Scale**	3.32 ± 1.04^b^	3.71 ± 0.80^b^	3.79 ± 0.92^a^	3.64 ± 1.04^b^	4.18 ± 1.14^a^	4.30 ± 1.01^a^	3.56 ± 1.13^b^	4.18 ± 1.21^a^	4.15 ± 0.78^a^
**Total faecal fibre [g/d]**	10.3 ± 4.5^b^	13.3 ± 5.5^a^	13.8 ± 9.3^a^	7.59 ± 3.73^b^	9.77 ± 4.85^a^	9.98 ± 4.3^a^	9.63 ± 3.79^b^	11.4 ± 4.4^ab^	13.5 ± 8.7^a^

Values are presented as mean ± standard deviation.

*BG* blue lupin group; *WG* white lupin group; *SG* soya group; blue lupin, *Lupinus angustifolius cv.* Boregine*;* white lupin, *Lupinus albus cv.* Typ Top*;* soya, *Glycine max cv.* Hefeng*;* citrus, citrus fibre Herbacel AQ Plus serving as active comparator.

^a>b>c^Significant differences between mean values within each group with unequal superscript letters (*P* ≤ 0.05) determined by repeated measures ANOVA.

^1^Oro-faecal transit time: BG (n = 24), WG (n = 20), SG (n = 20).

### Faecal composition and bowel habits

The lupin fibre supplementations increased the daily faecal dry matter and faecal weight compared to run-in, representing an increase of 1.76 g faeces/g additional dietary fibre contributed by blue lupin intervention and of 1.64 g faeces/g white lupin, respectively (Table 3). The relative proportion of dry matter in the faeces decreased significantly due to both lupin fibre interventions. The effects of soya on the faecal parameters were not significant. In all groups, daily faecal mass and faecal dry matter were significantly higher after intervention with citrus fibre compared to run-in but were not significantly different amongst the fibre supplementations within the groups. The relative proportion of dry matter decreased significantly due to citrus intervention, except in SG. Faecal pH value was found to decrease significantly in the blue lupin period compared to run-in (0.27 units, *P <* 0.01), but did not change compared to citrus. However, no significant differences between the study periods were seen in WG or SG. In BG, the oro-faecal transit time differed significantly between run-in and both fibre periods. Furthermore, as evaluated by the ‘Bristol Stool Form Scale’, appearance of faeces was significantly affected throughout the fibre interventions. In comparison to run-in, all fibre supplementations led to significantly higher stool form scores, which is equivalent to a looser and more watery faecal consistency. The appearance of faeces did not differ between fibre interventions within the groups.

For both interventions with lupin fibre, a significant enhancement of faecal excretion of total food-derived fibre was observed. In all groups, the consumption of citrus fibre resulted in a significantly higher faecal content of total food-derived fibre compared to run-in.

### Short-chain fatty acids

Both types of lupin interventions led to an enhanced formation of SCFA whereby total daily SCFA excretion as well as excretion of the main SCFA acetate, propionate, and *n*-butyrate increased significantly compared to run-in. No significant differences between the molar ratios (% of total) of the main SCFA during fibre interventions and run-in were observed in any group. Concerning the faecal concentrations of total SCFA, only blue lupin showed a significant enhancement compared to both the run-in (20%, *P* = 0.02) and citrus period (16%, *P* = 0.03). After blue lupin, the faecal concentrations of acetate and propionate were significantly increased compared to run-in. In comparison to citrus, the concentrations of propionate and *n*-butyrate were significantly higher after blue lupin. With regard to the faecal concentrations of the three main SCFA, no significant changes were observed following consumption of citrus fibre compared to run-in. All fibre interventions, particularly the citrus fibre, resulted in lower concentrations of *iso*-butyric acid and *iso*-valeric acid (Table 4).

**Table 4:** Concentration and daily excretion of short-chain fatty acids (SCFA) in faeces

	**BG (n = 26)**			**WG (n = 24)**			**SG (n = 26)**		
	**Run-in**	**Blue lupin**	**Citrus**	**Run-in**	**White lupin**	**Citrus**	**Run-in**	**Soya**	**Citrus**
**Faecal concentration [μmol/g faeces]**									
**Total SCFA**	62.9 ± 23.3^b^	75.4 ± 24.0^a^	65.3 ± 24.2^b^	69.4 ± 23.0	75.0 ± 21.4	72.4 ± 22.6	77.1 ± 21.1	81.3 ± 19.7	75.7 ± 23.5
**Acetate**	37.3 ± 13.1^b^	45.6 ± 12.3^a^	41.2 ± 15.9^ab^	41.5 ± 12.8	45.9 ± 12.5	45.0 ± 13.6	46.5 ± 12.4	49.6 ± 12.7	46.1 ± 14.4
**Propionate**	9.86 ± 4.73^b^	11.9 ± 5.0^a^	9.05 ± 3.89^b^	10.7 ± 5.3	11.9 ± 4.6	10.3 ± 4.3	13.2 ± 6.6^ab^	15.0 ± 4.9^a^	12.2 ± 5.5^b^
***iso*-Butyrate**	1.11 ± 0.45	0.97 ± 0.55	0.91 ± 0.49	1.49 ± 0.59^a^	1.25 ± 0.51^b^	1.15 ± 0.52^b^	1.25 ± 0.56^a^	1.21 ± 0.63^a^	0.95 ± 0.38^b^
***n*-Butyrate**	11.4 ± 6.5^ab^	14.0 ± 8.4^a^	11.3 ± 6.4^b^	11.0 ± 5.3	12.3 ± 6.3	11.9 ± 5.4	12.6 ± 5.1	12.1 ± 4.4	13.3 ± 6.3
***iso*-Valerat**	1.76 ± 0.70^a^	1.46 ± 0.78^b^	1.47 ± 0.79^b^	2.14 ± 0.89^a^	1.69 ± 0.90^b^	1.57 ± 0.77^b^	1.70 ± 0.88^a^	1.58 ± 1.02^ab^	1.28 ± 0.58^b^
***n*-Valerat**	1.17 ± 0.57	1.18 ± 0.60	0.98 ± 0.53	1.54 ± 0.72	1.41 ± 0.58	1.40 ± 0.61	1.29 ± 0.73	1.21 ± 0.78	1.14 ± 0.67
***n*-Caproate**^**W**^	0.26 ± 0.44^b^	0.30 ± 0.42^ab^	0.39 ± 0.50^a^	0.52 ± 0.67^b^	0.60 ± 0.57^ab^	0.99 ± 1.24^a^	0.58 ± 0.74	0.58 ± 0.77	0.66 ± 078
**Daily excretion [mmol/d]**									
**Total SCFA**	8.89 ± 6.30^b^	14.1 ± 9.5^a^	12.0 ± 10.0^a^	7.68 ± 5.78^b^	11.7 ± 9.1^a^	10.9 ± 7.2^a^	11.0 ± 6.8	12.9 ± 7.1	12.9 ± 6.5
**Acetate**	5.20 ± 3.53^b^	8.57 ± 5.95^a^	7.49 ± 6.18^a^	4.61 ± 3.49^b^	7.09 ± 5.50^a^	6.68 ± 4.10^a^	6.63 ± 4.23	7.96 ± 4.61	7.86 ± 4.13
**Propionate**	1.44 ± 1.20^b^	2.25 ± 1.61^a^	1.75 ± 1.70^b^	1.25 ± 1.08^b^	1.84 ± 1.47^a^	1.61 ± 1.38^ab^	1.91 ± 1.47	2.35 ± 1.25	2.11 ± 1.22
***iso*-Butyrate**	0.15 ± 0.08	0.15 ± 0.09	0.13 ± 0.08	0.14 ± 0.07	0.16 ± 0.09	0.16 ± 0.11	0.16 ± 0.09	0.17 ± 0.10	0.16 ± 0.08
***n*-Butyrate**	1.69 ± 1.45^b^	2.71 ± 2.35^a^	2.24 ± 2.21^a^	1.27 ± 1.15^b^	2.09 ± 2.01^a^	1.89 ± 1.48^a^	1.80 ± 1.28^b^	1.90 ± 1.24^b^	2.28 ± 1.48^a^
***iso*-Valerat**	0.22 ± 0.11	0.22 ± 0.12	0.20 ± 0.10	0.20 ± 0.10	0.21 ± 0.12	0.21 ± 0.13	0.21 ± 0.12	0.21 ± 0.11	0.21 ± 0.10
***n*-Valerat**	0.16 ± 0.13	0.19 ± 0.13	0.16 ± 0.13	0.16 ± 0.10	0.19 ± 0.11	0.20 ± 0.15	0.18 ± 0.13	0.18 ± 0.14	0.19 ± 0.13
***n*-Caproate**^**W**^	0.04 ± 0.08^b^	0.04 ± 0.06^ab^	0.05 ± 0.09^a^	0.05 ± 0.07^b^	0.08 ± 0.10^ab^	0.15 ± 0.22^a^	0.08 ± 0.11^b^	0.09 ± 0.13^ab^	0.11 ± 0.15^a^

Values are presented as mean ± standard deviation.

*BG* blue lupin group, *WG* white lupin group, *SG* soya group; blue lupin, *Lupinus angustifolius cv.* Boregine*;* white lupin, *Lupinus albus cv.* Typ Top*;* soya, *Glycine max cv.* Hefeng*;* citrus, citrus fibre Herbacel AQ Plus serving as active comparator.

^a>b>c^Significant differences between mean values within each group with unequal superscript letters (*P* ≤ 0.05) determined by repeated measures ANOVA.

^W^Values were not normally distributed; significance was calculated by means of the Wilcoxon test.

### Neutral sterols and bile acids

Low converter revealed a high cholesterol excretion together with a very low excretion of the microbial transformation product coprostanol. The reasons and outcomes of this phenomenon have not been sufficiently investigated because of the complexity of the host-microflora interactions [39]. Hence, the data were statistically analysed using the values after excluding low converters. A cholesterol conversion of ≤ 25% was defined as a cut-off level for classification as low converter. Eighteen participants (blue lupin: n = 7; white lupin: n = 3; soya: n = 8) showed this altered neutral sterol profile in at least one study period and were defined as low converters. After exclusion of low converters, the average daily excretion of neutral sterols remained constant over the entire study course (data not shown). However, the concentration of total sterols, coprostanol, and coprostanone in the dry faeces increased significantly under the blue lupin fibre-regimen compared to run-in (Table 5). Following citrus, the faecal concentration of coprostanone decreased significantly in white lupin compared to run-in.

**Table 5:** Concentration of neutral sterols and bile acids in dry faeces

**Faecal neutral sterol concentration [mg/g dry faeces]**									
	**BG (n = 19)**			**WG (n = 21)**			**SG (n = 18)**		
	**Run-in**	**Blue lupin**	**Citrus**	**Run-in**	**White lupin**	**Citrus**	**Run-in**	**Soya**	**Citrus**
**Total NS**	21.6 ± 8.1^a^	18.3 ± 5.3^b^	19.8 ± 6.6^ab^	22.8 ± 5.8	22.2 ± 6.3	20.8 ± 5.6	19.8 ± 5.8	18.7 ± 5.0	18.3 ± 5.4
**Cholesterol**	2.87 ± 1.82	2.56 ± 1.11	2.63 ± 1.67	2.54 ± 1.38	2.76 ± 1.53	2.98 ± 2.19	2.80 ± 2.30	2.19 ± 0.92	3.09 ± 1.62
**Coprostanol**	16.0 ± 6.5^a^	13.4 ± 4.6^b^	14.9 ± 5.5^ab^	16.8 ± 5.1	16.5 ± 5.7	15.3 ± 5.4	14.3 ± 5.3	14.2 ± 5.0	13.0 ± 5.0
**Cholestanol**	0.64 ± 0.11	0.64 ± 0.12	0.62 ± 0.15	0.56 ± 0.16	0.60 ± 0.08	0.59 ± 0.07	0.61 ± 0.10	0.61 ± 0.14	0.60 ± 0.12
**Coprostanone**	1.85 ± 1.32^a^	1.38 ± 1.00^b^	1.40 ± 0.81^ab^	2.49 ± 1.55^a^	1.94 ± 1.23^ab^	1.57 ± 0.85^b^	1.67 ± 0.88	1.31 ± 0.75	1.26 ± 0.62
**Cholestanone**	0.04 ± 0.06	0.05 ± 0.05	0.05 ± 0.06	0.14 ± 0.03	0.14 ± 0.06	0.13 ± 0.03	0.10 ± 0.03	0.11 ± 0.02	0.11 ± 0.04
**Cholestenone**	0.24 ± 0.14	0.20 ± 0.11	0.22 ± 0.11	0.30 ± 0.08	0.27 ± 0.07	0.30 ± 0.12	0.26 ± 0.09	0.24 ± 0.07	0.27 ± 0.11
**Faecal bile acid concentration [mg/g dry faeces]**									
	**BG (n = 26)**			**WG (n = 24)**			**SG (n = 25)**		
	**Run-in**	**Blue lupin**	**Citrus**	**Run-in**	**White lupin**	**Citrus**	**Run-in**	**Soya**	**Citrus**
**Total BA**	6.97 ± 2.72^a^	6.03 ± 2.17^a^	5.07 ± 1.82^b^	6.86 ± 1.86^a^	5.45 ± 1.73^b^	5.27 ± 1.95^b^	6.40 ± 2.39^a^	5.33 ± 2.49^b^	5.39 ± 2.28^b^
**Primary BA**	0.53 ± 0.27	0.66 ± 0.46	0.51 ± 0.29	0.53 ± 0.34	0.62 ± 0.61	0.42 ± 0.15	0.57 ± 0.28^a^	0.44 ± 0.22^b^	0.51 ± 0.30^ab^
**Cholic acid**	0.28 ± 0.15	0.35 ± 0.25	0.26 ± 0.15	0.28 ± 0.16	0.36 ± 0.41	0.24 ± 0.13	0.28 ± 0.12^a^	0.22 ± 0.11^b^	0.26 ± 0.17^ab^
**Chenodeoxycholic acid**	0.26 ± 0.14	0.31 ± 0.23	0.24 ± 0.16	0.25 ± 0.27	0.25 ± 0.22	0.18 ± 0.07	0.30 ± 0.18^a^	0.22 ± 0.13^b^	0.25 ± 0.14^ab^
**Secondary BA**	6.43 ± 2.68^a^	5.36 ± 2.17^b^	4.57 ± 1.69^b^	6.33 ± 1.77^a^	4.83 ± 1.84^b^	4.85 ± 1.91^b^	5.83 ± 2.26^a^	4.89 ± 2.37^ab^	4.88 ± 2.23^b^
***Iso*-lithocholic acid**	1.15 ± 0.56^a^	0.96 ± 0.44^b^	0.91 ± 0.48^b^	0.99 ± 0.42^a^	0.80 ± 0.46^b^	0.88 ± 0.51^ab^	1.00 ± 0.47	1.02 ± 0.50	0.90 ± 0.55
**Lithocholic acid**	1.57 ± 0.79^a^	1.27 ± 0.69^b^	1.06 ± 0.53^c^	1.45 ± 0.51^a^	1.12 ± 0.36^b^	1.03 ± 0.37^b^	1.38 ± 0.58^a^	1.08 ± 0.51^b^	1.07 ± 0.47^b^
***Iso*-deoxycholic acid**	1.04 ± 0.65^a^	0.91 ± 0.55^ab^	0.78 ± 0.42^b^	1.10 ± 0.67^a^	0.86 ± 0.64^b^	0.93 ± 0.74^ab^	1.13 ± 0.56	1.12 ± 0.76	1.03 ± 0.66
**Deoxycholic acid**	2.43 ± 1.30^a^	2.03 ± 0.95^a^	1.65 ± 0.88^b^	2.53 ± 1.03^a^	1.89 ± 0.97^b^	1.85 ± 0.87^b^	2.13 ± 1.12^a^	1.51 ± 1.03^b^	1.70 ± 1.02^b^
**12nketodeoxycholic acid**	0.23 ± 0.15^a^	0.20 ± 0.17^ab^	0.17 ± 0.09^b^	0.26 ± 0.24^a^	0.16 ± 0.06^b^	0.16 ± 0.06^b^	0.19 ± 0.07	0.16 ± 0.07	0.18 ± 0.09

Values are presented as mean ± standard deviation.

*BG* blue lupin group, *WG* white lupin group, *SG* soya group; blue lupin, *Lupinus angustifolius cv.* Boregine*;* white lupin, *Lupinus albus cv.* Typ Top*;* soya, *Glycine max cv.* Hefeng*;* citrus, citrus fibre Herbacel AQ Plus serving as active comparator; *NS* neutral sterols, *BA* bile acids.

^a>b>c^Significant differences between mean values within each group with unequal superscript letters (*P* ≤ 0.05) determined by repeated measures ANOVA.

In the faeces, blue lupin fibre intervention significantly increased the daily excretion of the sum of primary bile acids (18.2 ± 12.1 *vs.* 27.9 ± 22.5 mg/d, *P* = 0.02) as well as cholic acid (9.26 ± 5.46 *vs.* 14.6 ± 12.0 mg/d, *P =* 0.02) and chenodeoxycholic acid (8.98 ± 7.32 *vs.* 13.3 ± 11.1 mg/d, *P =* 0.03) compared to run-in. The other fibre interventions did not alter excretion of bile acids (data not shown).

All three interventions with legume fibre reduced the concentration of total bile acids (blue lupin: 14%, *P =* 0.05; white lupin: 21%, *P* < 0.01; soya: 17%, *P =* 0.04) and the sum of secondary bile acids (blue lupin: 16%, *P =* 0.03; white lupin: 24%, *P* < 0.01; soya: 16%, *P =* 0.06) in dry faeces. Due to supplementation with citrus fibre, the concentration of total bile acids (mean: 22%, *P* < 0.03) and secondary bile acids (mean: 23%, *P* < 0.03) decreased significantly in comparison to run-in in all groups. The changes in the concentrations can mainly be ascribed to the decrease of lithocholic acid and deoxycholic acid (Table 5). Soya was the only fibre that decreased the sum of primary bile acids (23%, *P =* 0.04) as well as cholic acid (21%, *P =* 0.04) and chenodeoxycholic acid (27%, *P =* 0.04) in dry faeces.

### Blood lipids

Total cholesterol, HDL cholesterol, LDL cholesterol, and triacylglycerol concentrations in serum were not influenced by any of the interventions (data not shown). All values determined from the participants were within the normal range (total cholesterol: 4.84 ± 1.04 mmol/L; HDL cholesterol: 1.57 ± 0.44 mmol/L; LDL cholesterol: 2.67 ± 0.73 mmol/L; triacylglyceroles: 1.09 ± 0.44 mmol/L; Table 1).

## Discussion

Administration of the tested lupin and citrus fibre at a dose of 25 g/day for two weeks resulted in an increase in daily faecal mass and faecal dry matter. Increased faecal bulking and, therefore, dilution of carcinogens is one of the proposed mechanisms by which dietary fibre reduce the risk of colorectal cancer [16,24]. In contrast to the lupin interventions, the effects of soya intervention on faecal mass and faecal excretion of total food-derived fibre were not significant. Other studies detected more distinct effects for soya fibre regarding faecal bulking [18]. Because both lupin fibres contain less quantities of water-insoluble fibre than soya, fermentable fibre fractions must be responsible for the enhanced faecal mass due to a gain in bacterial mass. Moreover, lupin kernel fibre is characterised by a higher water-binding capacity [20,40]. A daily faecal mass > 150 g/d is supposed to lower the risk of colorectal cancer [24]. We observed that consumption of blue lupin and soya, as well as citrus in these respective groups, resulted in an increase in mean faecal weight above this low-risk cut-off value. The higher gut content stimulates intestinal peristalsis reducing the oro-faecal transit time [15,24]. In the present study, the supplementation with blue lupin led to the highest increase in faecal weight (33%, *P* < 0.01) and shortened the oro-faecal transit time significantly by 21% (*P =* 0.04). Moreover, subjects with a long transit time, especially participants suffering from constipation, reported an increased frequency of defaecation and an improved ease of defaecation confirmed by a meliorated faecal consistency. The obtained results are in line with the findings of a study investigating the effects of a high-fibre diet containing lupin kernel fibre (*L. angustifolius*) by Johnson *et al.*[19]. The intake of 17 - 30 g/d lupin kernel fibre for four weeks led to an increase of 21% in faecal output and resulted in a 17% decline in transit time [19].

In the current study, all tested fibre interventions led to an enhanced excretion of total SCFA as well as the main SCFA acetate, propionate, and *n*-butyrate (Table 4). However, a significant increase could only be shown as a consequence of intervention with both lupin fibres, which contain a higher content of water soluble fibre than soya and citrus fibre. The study by Johnson *et al.* provided the first evidence that lupin kernel fibre modifies levels of faecal SCFA [19]. The present data revealed that SCFA concentration was not affected to a large extent because the SCFA formed were diluted due to the increased faecal mass. SCFA, particularly *n*-butyrate, are an important energy source for colonocytes. In addition, *n*-butyrate is able to reduce the risk of malignant changes through regulation of colonocyte differentiation [25,41,42]. Due to the enhanced SCFA formation, both lupin fibre interventions could elevate *n*-butyrate excretion significantly (blue lupin: 60%, *P* < 0.01; white lupin: 65%, *P* < 0.01) and the faecal concentration slightly (blue lupin: 23%, *P* = 0.11; white lupin: 12%, *P* = 0.18). Increased SCFA concentration lowers the pH value, which consequently decreases the formation of carcinogenic substances [24,27]. A significant negative correlation (r = -0.58(^s^), *P* < 0.01) of SCFA excretion and faecal pH confirms the influence of SCFA on pH value in colon and faeces. In the present study, a significantly lower faecal pH was observed after blue lupin intervention. The reduction in the faecal pH values after blue lupin was of equal magnitude to that previously observed for lupin kernel fibre diet [19]. Despite the observed changes in SCFA excretion after white lupin intervention, faecal pH was not reduced (Table 3).

All types of fibre used in this study had no effect on the daily excretion of neutral steroids which is similar to results obtained from previous studies of other types of dietary fibre [15]. The concentrations of total neutral sterols, coprostanol, and coprostanone decreased after intervention with blue lupin as a result of the dilution effect due to the increased faecal mass.

Only blue lupin increased the faecal excretion of primary bile acids, whereas the average excretion of total bile acids remained unchanged. It appears that contrary to what is reported in the literature and demonstrated *in vitro*[31], bile acids were not bound by the fibre preparations. In fact, the decrease in the pH value seems to be responsible for the increased excretion of primary bile acids. A pH value < 6.5 (blue lupin: 6.38) inhibits the activity of 7 α-dehydroxylase, which is involved in the conversion of bile acids and enhances the precipitation of bile acids [4]. Moreover, all fibre interventions lowered the faecal concentration of bile acids (Table 5). Since the excretion of bile acids remained unchanged, this decrease in concentration is due to the increase of daily faecal mass or dry matter and the altered pH value. Acid steroids, particularly the secondary bile acids, are potential risk factors for colorectal cancer [22,28,29]. Indeed, various studies have shown that bile acids play an etiologic role in colorectal cancer by causing DNA damage. Numerous epidemiological studies support the hypothesis that incidence of colorectal cancer correlates with faecal bile acid levels [28]. Furthermore, the modified milieu in the colon might restrict growth of potential pathogens as well as putrefactive bacteria. A study in 18 male subjects ingesting 17–30 g/d lupin kernel fibre incorporated in food for a period of 28 days revealed that lupin kernel fibre may be considered as prebiotic since colonic bifidobacteria growth was found stimulated [43].

A deficit of bile acids in the enterohepatic circulation should lead to a compensatory synthesis of new bile acids from cholesterol in the liver which, in turn, results lower blood cholesterol concentrations [30]. As mentioned above, bile acid-binding was not detected in the present study, because the fibre interventions did not alter the excretion of total bile acids. In line with this, blood cholesterol levels remained unaffected. In contrast to our results, one study, which focused on foods enriched with lupin kernel fibre, revealed that additional consumption of 17 - 30 g/d lupin fibre for one month resulted in significant reductions of total and LDL cholesterol compared to the controlled diet. Hall *et al*. underlined that the cholesterol-lowering effect was restricted to hypercholesterolaemic subjects [34]. In another study of our work group in hypercholesterolaemic subjects, a four-week intervention with 25 g/d blue lupin incorporated in food led also to a decrease of total and LDL cholesterol, in spite of unchanged bile acid excretion [21]. Hence, the below average cholesterol concentration in the present study (Table 2) may explain the lack of effect of the tested fibre preparations on blood lipids.

## Conclusions

The findings of the present study confirm that dietary fibre from different plant sources often demonstrate unique physiological effects in the gut, which is in line with the inconsistent data achieved from a number of studies investigating an association between dietary fibre consumption and occurrence of colorectal cancer. In addition, other aspects such as fat content in the diet, consumption of red meat, and presence of micronutrients may also play a role in the development of colorectal cancer. Hence, randomised controlled intervention studies are not able to directly establish the effects of an increased dietary fibre intake on the development of colorectal cancer [44]. In line with this, a Cochrane meta-analysis of five randomised controlled trials (in a total of 4349 subjects) of increased dietary fibre intake to prevent recurrence of adenomatous polyps, as surrogate endpoint, found no difference between intervention and control groups with regard to the development of adenomas [45]. Nevertheless, the randomised controlled trial selected for the current analysis is the most appropriate study design to investigate specific types of fibres as well as to interpret and compare their individual effects, at least for the respective study population.

**Figure 3:**
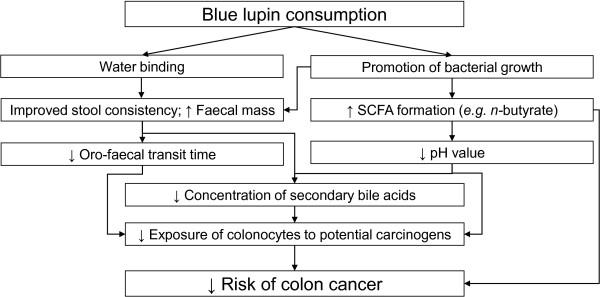
**Schematic summary of the physiological effects of blue lupin fibre in the colon**[21]**.** blue lupin, *Lupinus angustifolius cv.* Boregine.

Results obtained in the present study show that a daily intake of 25 g legume kernel fibre or citrus fibre over two weeks failed to affect lipid metabolism through bile acid-binding in healthy, normocholesterolaemic subjects. However, the obtained findings suggest that all tested fibre preparations might be able to prevent constipation as they improve faecal consistency and oro-faecal transit time and therefore exert a positive impact on colonic function. The fibre preparations of legumes, particularly *Lupinus angustifolius cv.* Boregine additionally have beneficial effects on putative risk factors for colorectal cancer such as daily faecal mass, oro-faecal transit time, SCFA production, pH value, and concentration of secondary bile acids (Figure 3).

Overall, it can be concluded that increasing dietary fibre intake of blue lupin kernel fibre in the range of about 50 g/d may in general and, in particular, in predisposed people contribute towards the prevention of colorectal cancer and support medical therapies.

## Abbreviations

Blue lupin: Blue lupin kernel fibre (*Lupinus angustifolius cv.* Boregine); White lupin: White lupin kernel fibre (*Lupinus albus cv.* Typ Top); Soya: Soya bean kernel fibre (*Glycine max cv.* Hefeng); Citrus: Citrus fibre (Herbacel AQ Plus); BG: Blue lupin group; WG: White lupin group; SG: Soya group; TDF: Total dietary fibre; SCFA: Short-chain fatty acids.

## Competing interests

The authors declare that they have no personal or financial competing interests.

## Authors’ contributions

AF and GJ were responsible for conception and study design; AF was responsible for the supervision of the human study, sample handling, coordination and conduction of the analyses, and statistical analysis; KF carried out bile acid analyses; AF and GJ were responsible for data interpretation; AF wrote the paper; all authors read and approved the final manuscript.

## Supplementary Material

Additional file 1 CONSORT 2010 checklist of information to include when reporting a randomised trial*. Click here for file
